# Naringin-based hydrogel decorated 3D printed scaffold modulates immunity and angiogenesis for diabetic bone regeneration

**DOI:** 10.1093/rb/rbag161

**Published:** 2026-08-05

**Authors:** Zecai Chen, Peng Luo, Lei Qin, Zhen Xu, Zhenqian Qi, Yingjie Ma, Zheng Xiao, Jiarui Fang, Shuo Tang, Dazhi Yang

**Affiliations:** Department of Spine Surgery, Affiliated Nanshan Hospital of Shenzhen University, Shenzhen 518052, China; Department of Sports Medicine, Affiliated Nanshan Hospital of Shenzhen University, Shenzhen 518052, China; Department of Spine Surgery, Affiliated Nanshan Hospital of Shenzhen University, Shenzhen 518052, China; Department of Spine Surgery, Affiliated Nanshan Hospital of Shenzhen University, Shenzhen 518052, China; Department of Spine Surgery, Affiliated Nanshan Hospital of Shenzhen University, Shenzhen 518052, China; Department of Spine Surgery, Affiliated Nanshan Hospital of Shenzhen University, Shenzhen 518052, China; Department of Spine Surgery, Affiliated Nanshan Hospital of Shenzhen University, Shenzhen 518052, China; Department of Sports Medicine, Affiliated Nanshan Hospital of Shenzhen University, Shenzhen 518052, China; Department of Orthopedics, The 8th Affiliated Hospital of Sun Yat-Sen University, Shenzhen 518052, China; Department of Spine Surgery, Affiliated Nanshan Hospital of Shenzhen University, Shenzhen 518052, China

**Keywords:** diabetic bone healing, naringin, 3D-printed, angiogenesis, immunomodulation, osteogenesis

## Abstract

Hyperglycemia and inflammation within the diabetic microenvironment impair the healing of bone defects in diabetic patients. A key limitation of current therapeutic approaches is their lack of efficacy in restoring immune homeostasis. Herein, a novel 3D-printed PHSN composite scaffold is designed to regulate the immune microenvironment. PHSN is fabricated by integrating poly (lactic-co-glycolic acid) (PLGA) with hydroxyapatite (HA) via 3D printing technology, yielding a structure that combines osteogenic potential with mechanical strength. Loaded with naringin and SupGels, PHSN promotes macrophage repolarization toward the M2 phenotype via the JAK/STAT signaling pathway, thereby upregulating anti-inflammatory mediators and tissue-regeneration factors, stimulating angiogenesis and osteogenesis. In diabetic models, PHSN inhibits M1 macrophage polarization, drives reprogramming toward the M2 phenotype and upregulates the expression of CD31, ALP and OCN at bone defect sites, indicating enhanced angiogenesis and osteogenesis. Collectively, this study establishes a strategy of synergistically modulating immunity, vascularization and bone formation, offering a promising and translatable solution for diabetic bone regeneration.

## Graphical abstract

**Graphical Abstract:**
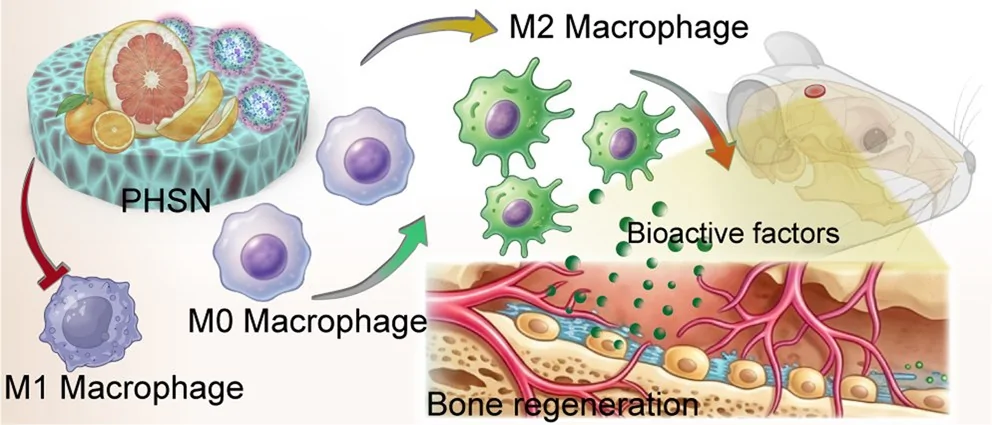

## Introduction

Bone defects are major clinical challenges in modern medicine, and approximately 5–10% of patients experience delayed healing or nonunion, with large bone defects having an almost 90% nonunion rate [1]. Although native bone is endowed with intrinsic regenerative capacity, in the comorbid conditions of osteoporosis, diabetes, wound infection and periodontitis, this physiological bone-healing is significantly impaired [2]. Diabetes mellitus (DM) is a major chronic metabolic disorder both in China and globally, and the global prevalence of diabetes is projected to exceed 10% by 2030 [3]. Diabetes is characterized by hyperglycemia, chronic oxidative stress and inflammation [4]. Patients with diabetes mellitus exhibit severe low bone mass, decreased bone mineral density, reduced trabecular number and degenerated bone microstructural morphologies, increasing fracture risk and impairing healing of bone defects [5]. Notably, in diabetic bone defects, clinical evidence demonstrated that diabetic patients exhibit a 2.11-fold higher risk of compromised fracture healing relative to non-diabetic individuals [6]. In mechanistic studies, the diabetic hyperglycemic microenvironment inhibits vascular regeneration and promotes inflammation [7, 8]. Under inflammatory conditions, macrophages polarize toward the pro‑inflammatory M1 phenotype at bone defect sites, accompanied by elevated reactive oxygen species (ROS) levels. Excessive ROS accumulation coupled with exposed wound tissues is extremely susceptible to bacterial infection, which further impairs bone repair [9–11]. Currently, immune microenvironment regulation, reactive oxygen species (ROS) scavenging and angio-osteogenic coupling are the major therapeutic strategies [12]. Regulating macrophage polarization is the core strategy to remodulate the inflamed microenvironment of diabetic bone defects. The emergence of numerous novel biomaterials, including smart and dynamic hydrogel systems [13, 14], vascularized 3D-printed scaffolds [15] and functionalized polyetheretherketone (PEEK) implants [16], can effectively suppress M1 macrophage polarization, facilitate M2 macrophage polarization, stimulate angiogenesis and enhance the bone regenerative efficacy of diabetic bone defects. Therefore, it is significant to develop effective treatment strategies to regulate the diabetic bone microenvironment for promoting diabetic bone regeneration.

Naringin, a natural flavanone, is characterized by its anti-inflammatory and anti-osteoporotic bioactivities [17]. Naringin can notably suppress the levels of TNF-α, IL-1β and IL-6 [18], thereby promoting the M2 macrophage phenotype [19]. Furthermore, naringin reduces LPS-induced pro-inflammatory responses by suppressing NF-κB activation and the p38/MAPK signaling pathway [20]. Some studies have shown that naringin-loaded microspheres or naringin-loaded hydrogel promote M2 macrophage polarization through JAK1/STAT6, AMPK/PGC-1α or PPARα/STING signaling pathways [21–23]. However, the downstream effects following M2 polarization regulation remain unclear. Polylactide-co-glycolide (PLGA) has been extensively utilized as a bone scaffold material in bone tissue engineering. It not only possesses easy availability, favorable biocompatibility, low cytotoxicity, high porosity and tunable degradation rate, but also serves as a versatile carrier for drug delivery [24]. Combining hydroxyapatite (HA) nanoparticles with PLGA further enhances the osteogenic activity of the composite [25]. However, the unmodified PLGA/HA composite lacks bioactivity and immunomodulatory capacity, which renders it unable to meet the requirements for treating complicated diabetic bone defects. Supramolecular hydrogels (SupGels) are synthesized from gelatin and photopolymerizable acrylated β-cyclodextrin (β-CD) monomers [26]. SupGels exhibit rapid self-healing properties, efficiently promote cell recruitment and differentiation and possess excellent drug delivery capacity [27, 28]. SupGels enhance angiogenesis and facilitate human blood vessel differentiation into arterioles containing smooth muscle cells [29]. However, the SupGels exhibit weak mechanical properties and cannot provide adequate mechanical support to the bone defect. Therefore, the incorporation of naringin and SupGels into the PLGA/HA scaffold is expected to yield a composite with excellent immunomodulatory, angiogenic and osteogenic properties, offering a potential strategy for the repair of diabetic bone defects.

In this study, a novel PHSN (PLGA/HA/SupGels/Naringin) composite scaffold was fabricated using 3D printing technology, and its osteogenic capacity during the healing process of diabetic bone defects was systematically evaluated (Figure 1). Specifically, the rigid PLGA/HA scaffolds were fabricated to mimic the porous structure of bone, thereby promoting new bone ingrowth. Meanwhile, the soft hydrogel matrix loaded with naringin was employed to decorate the scaffold. This configuration exhibits excellent immunoregulatory properties within the microenvironment, promoting bone regeneration in diabetic bone defects and thereby representing a promising therapeutic strategy.

**Figure 1:**
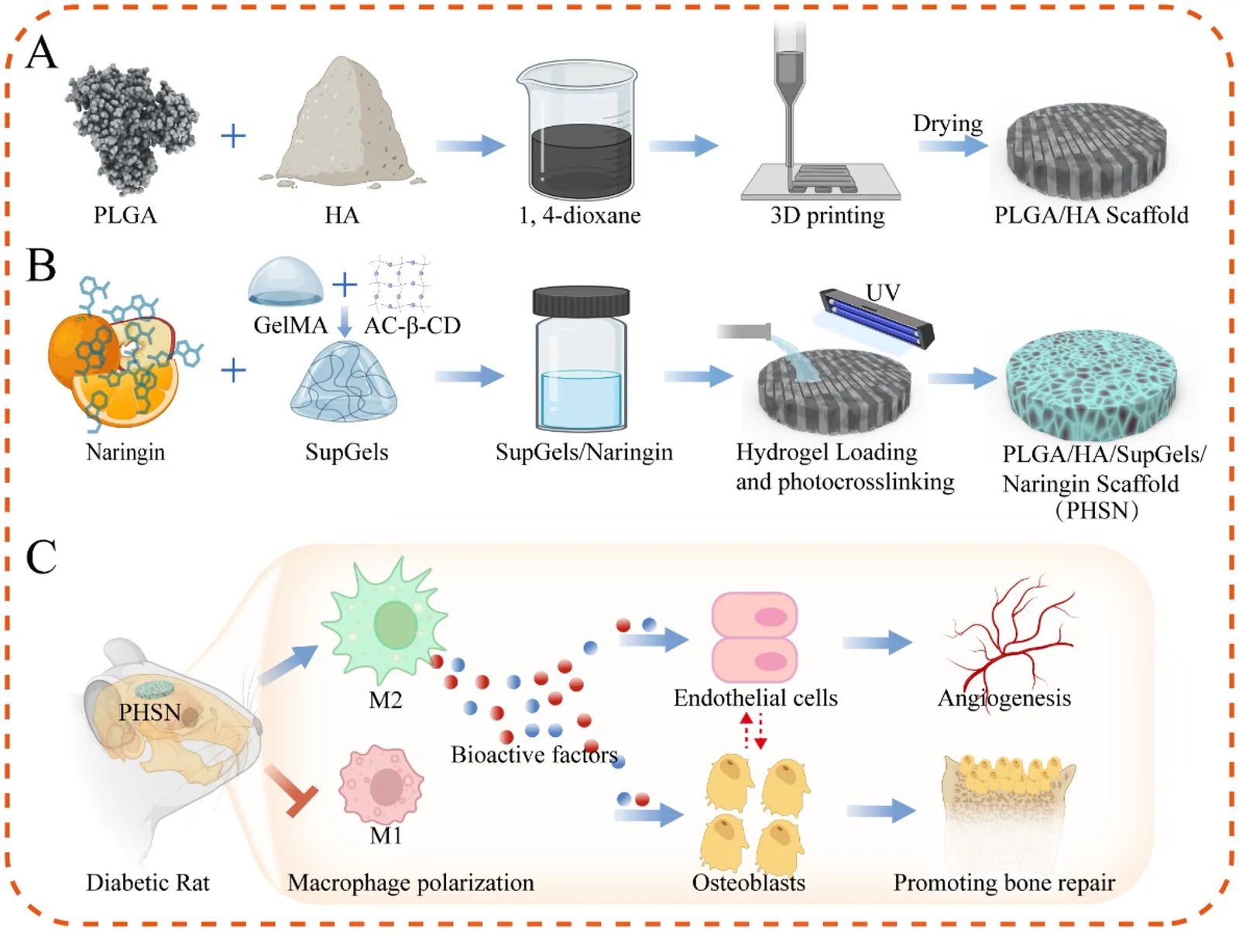
Schematic representation of the PHSN scaffold and the regulatory mechanisms of diabetic bone defect regeneration. (**A**) The fabrication of PLGA/HA scaffold. (**B**) Functional modification of the PLGA/HA scaffold. (**C**) The bone repair effect of PHSN scaffold *in vivo*. Schematic illustration created with adobe illustrator. PLGA, poly (lactic-co-glycolic acid); HA, hydroxyapatite; GelMA, gelatin methacrylamide; AC-β-CD, acrylated β-cyclodextrin; SupGels, supramolecular hydrogels consisting of GelMA and AC-β-CD; PHSN, PLGA/HA/SupGels/naringin scaffold.

## Materials and methods

### Preparation of 3D printed scaffolds

The PLGA (75:25, BIOMQ, Shenzhen, China) was solubilized in 1,4-dioxane (Aladdin, China) to yield a homogeneous 10% (w/v) solution. Subsequently, HA (Hydroxyapatite, Mqsw210301, BIOMQ, Shenzhen, China) was added into the PLGA solution, resulting in a final mass ratio of 1:4 (HA: PLGA, w/w). The PLGA/HA mixture was then homogenized with a magnetic stirrer until a consistent liquid paste was obtained. These scaffolds were fabricated by a 3D printing system (Bio-Architect@ WS, REGENOVO, Hangzhou, China), a 27 G nozzle was adopted, the print speed (45 mm s^−1^), pressure (0.1 MPa) and platform temperature (−20°C) were applied, respectively. Subsequently, these scaffolds were lyophilized using a laboratory freeze dryer (Bo Yi Kang FD-1-50, Beijing, China) at a vacuum pressure of 20–40 Pa for 24 h. The resulting scaffolds, composed of PLGA and HA, were termed PH.

Ten grams of β-cyclodextrin (β-CD, HY-107201, MCE, USA) were dissolved in 150 mL of dimethylformamide (DMF) (Fisher Scientific, NJ, USA), and then 7 mL of triethylamine (TEA, 81101, Merck, USA) was added to the reaction mixture. The mixture was stirred and cooled to 0°C. Then, 5 mL acrylic acid (306215, Merck, USA) was dropwise introduced. Following a 12-hour stirring period, the mixture was to remove excess TEA. The filtrate was concentrated to approximately 20 mL using a rotary evaporator under vacuum. The concentrated solution was added dropwise into 600 mL of acetone, which precipitated the modified cyclodextrin, thereby yielding AC-β-CD. Additionally, 10 g of gelatin (1288485, Merck, USA) was introduced into 100 mL PBS maintained at 50°C, and then was added 12 mL of methacrylic anhydride (276685, Merck, USA) to the 10% gelatin solution, and the reaction mixture was stirred at 50°C for 4 h. In order to remove unreacted reagents, the mixture was purified by dialysis against deionized water for over a period of 7 days at 45°C, using a dialysis membrane (SP132645, OriLeaf, Shanghai, China) with a 6 kDa cut-off, and then the gelatin was obtained by lyophilization at −104°C for 96 h. Finally, gelatin and AC-β-CD were mixed into PBS at 37°C to prepare mixed solutions with a fixed concentration of 8% (w/v) and 10% (w/v). Subsequently, the photoinitiator (I2959, MCE, NJ, USA) was added at 0.05% (w/v) to yield the SupGels.

All the scaffolds were washed with 100% ethanol and PBS, respectively, with each solvent applied for three cycles. Naringin (HY-N0119, MCE, NJ, USA) was prepared in 1 mL of PBS to obtain a final concentration of 100 μg/mL. The SupGels were then dispersed in 1 mL of naringin solution to create a naringin-SupGels mixture. Subsequently, the 3D-printed scaffolds were decorated with this mixture. Finally, these scaffolds were exposed to UV light (365 nm) for 5 min to facilitate crosslinking and gelation. These treated scaffolds were designated PHSN. In contrast, scaffolds decorated with naringin-free SupGels were labeled as PHS. Subsequently, the scaffolds underwent three PBS washes to remove crosslinking agent residues. Finally, the scaffolds were freeze-dried again and maintained at −20°C until needed.

### Characterization of 3D printed scaffolds

Firstly, these scaffolds were sputtered with a thin layer of gold coating, and then a scanning electron microscope (SEM, FEI Quanta 200, Japan) with energy dispersive spectroscopy (EDS) was used to observe the morphology of the scaffolds. Subsequently, an attenuated total reflectance Fourier-transform infrared (ATR-FTIR, Lambda, Tianjin, China) spectrometer was used to analyze the chemical structure of the dried microporous scaffolds. Spectra were acquired across the 1000–4000 cm^−1^ range, accumulating 32 scans using a diamond crystal smart Orbit ATR accessory Omnic 8.0 software. The cumulative release profile for naringin was determined by UV spectrophotometry. The inductively coupled plasma optical emission spectrometer (ICP-OES, Agilent 7700, USA) was used to quantify the concentrations of calcium and phosphorus. In order to evaluate the surface hydrophilicity, all scaffolds from each group were washed with deionized water, and then the water contact angles were measured by a scientific instrument (SCI3000F, Huanqiu Henda Technology, Beijing, China). The samples (5 × 10 × 10 mm^3^) were incubated at 37°C on days 1, 3, 5, 7, 9, 11, 13 and 15; the scaffold degradation rates were assessed using the following equation:


Rate of degradation (%)=Mt/M0×100%

Where *M_0_* and *M_t_* represent the initial scaffold mass and the mass at specified time points, respectively.

### Naringin release behavior of 3D printed scaffolds

The naringin release profile of the PHSN scaffold was assessed using PBS at a pH of 7.5. The PHSN scaffold was placed in the release medium (PBS) and kept at 37°C. At predetermined time points (days 1, 3, 5, 7, 9, 11, 13 and 15), 0.5 mL of the release medium was removed and replenished with an equal volume of fresh medium. The concentration of naringin was determined by UV spectroscopy with the aid of an analytical calibration curve.

### Cell culture

SD-BMSCs were sourced from Shanghai Zhong Qiao Xin Zhou Biotechnology (ZQY122, CELL RESEARCH, Shanghai, China). The SD-BMSCs were cultured in DMEM medium (DMEM; HyClone, USA) containing 10% FBS (Gibco, New York, USA) and 1% penicillin-streptomycin (P/S) (Yeasen Biotech, Shanghai, China). HUVECs were sourced from Procell Life Science and Technology (CL-0675; Wuhan, China) and cultured in the specific growth medium for PUMC-HUVEC-T1 (Pricella Life Science & Technology Co., Ltd, CM-0675). Mouse RAW264.7 cells were sourced from Procell Life Science & Technology (CL-0190; Wuhan, China) and cultured in DMEM (High-glucose, 4.5 g L^−1^ D-glucose; HyClone, UT, USA) supplemented with 10% FBS and 1% P/S. All cells were cultured at 37.5°C in a 5% CO_2_ environment, with the medium changed every 3 days.

### Cell viability assay

In order to detect the biological activity of cells, seven groups of microporous scaffolds with gradient naringin concentrations of 10, 50, 100, 150, 200, 250, 300 μg/mL were prepared. Bone marrow-derived stem cells (BMSCs) were seeded into 96-well plates at 5 × 10^4^ cells/mL, followed by the addition of scaffolds with different naringin concentrations to each well. CCK-8 (C0041, Beyotime, Shanghai, China) reagents (10%) were added on days 0, 1, 2 and 3. Plates were incubated at 37°C in the dark, and the optical density (OD) at 450 nm was measured.

### Cell cytotoxicity of 3D printed scaffolds

To investigate the potential cytotoxicity of the scaffolds, a co-culture system comprising the scaffolds and BMSCs was maintained for 24 h in a cell incubator (37°C, 5% CO_2_) to further assess the biological effects of the scaffolds. Live/Dead (#C2015S, Beyotime, Shanghai, China) staining was performed, and the samples were imaged with a confocal laser scanning microscope (CLSM, FV3000, Olympus, Tokyo, Japan). The proportion of dead cells to live cells was quantified as an indicator to compare the viability of BMSCs. Concurrently, Phalloidin staining (1:1000, #PF00001, Proteintech, Wuhan, China) was performed to examine the cell morphology on the scaffolds.

### Inflammatory response of 3D printed scaffolds

In order to assess the inflammatory response of the macrophages, different scaffolds were placed into the upper Transwell chamber (Millipore, Billerica, USA) plate; subsequently, RAW264.7 cells were plated in the basal chamber of the Transwell system at a density of 1.5 × 10^5^ cells/well and cultured for 48 h prior to the assay. Following fixation and permeabilization using 4% paraformaldehyde (P0099, Beyotime, Shanghai, China) and 0.1% Triton-X-100 (9002-93-1, Solarbio, Beijing, China) for 15 min and then blocking with 3% BSA serum (ST023, Beyotime, Shanghai, China) for 1 h, RAW264.7 cells were treated overnight at 4°C in the dark with specific primary antibodies for the CD206 (mannose receptor) (CD206, ab300621, Abcam), iNOS (inducible nitric oxide synthase) (iNOS, ab178945, Abcam) and F4/80 (ab6640, Abcam). Subsequently, the cells were incubated in the dark for 1 h at room temperature with secondary antibodies, specifically Alexa Fluor 488 goat anti-rabbit (4416 s, CST) and Alexa Fluor 594 goat anti-rat (8889 s, CST). Finally, DAPI staining solution was used to stain the cell nuclei, and after sealing with an anti-fluorescence quenching coverslip, the immunofluorescence images were visualized by confocal laser scanning microscopy and quantified using ImageJ 1.8.0 software.

### Tube formation assay

Different scaffolds were loaded into the upper Transwell chamber, while RAW264.7 cells were seeded in the lower Transwell chamber at 1.5 × 10^5^ cells/well. After 48 h, the cell medium from the lower chamber was collected and designated as conditioned medium. HUVEC cells were plated onto a Matrigel-coated 6-well plate at a density of 3 × 10^4^ cells per well (CLS354234, Corning, USA). The conditioned medium and DMEM medium were mixed at a 1:1 ratio, and the mixture was then added to the 6-well plate for 8 h. Finally, images were acquired under an inverted bright-field microscope (Nikon, ECLIPSE, Tokyo, Japan).

### Cell migration assay

A scratch-wound assay was performed to quantify the migratory capacity of cells. HUVEC cells were seeded in a 6-well plate and cultured at a density of 1.5 × 10^5^ cells per well until 90% confluency was achieved. A straight-line scratch was created using a 200 µL pipette; the culture medium was discarded and washed three times with PBS. Subsequently, the cells were treated with the conditioned medium for 12 and 24 h, respectively. Photographs were taken under a microscope at predetermined time points. The images were analyzed by ImageJ 18.0 software.

### Osteogenesis assay

Different scaffolds were placed in the upper Transwell chamber, while RAW264.7 cells were plated in the lower Transwell chamber at a density of 1.5 × 10^5^ cells per well. After 48 h, the cell medium from the lower chamber was collected and designated as conditioned medium. SD-BMSCs were plated at a density of 2 × 10^5^ cells in the bottom plate and maintained in osteogenic induction medium (RAXMX-90021, OriCell, China) for 7 and 21 days, respectively. Subsequently, mineralization was evaluated via ALP (P0321S, Beyotime, China) at 7 days and Alizarin Red (ALIR-10001, OriCell, Suzhou, China) at 21 days. Bright-field images were acquired under an inverted microscope.

### Transcriptome sequencing

To investigate the key molecular mechanisms underlying PHSN scaffold-mediated macrophage polarization, RNA sequencing was performed on RAW264.7 cells co-cultured with PHSN scaffolds for 48 h and compared with a control group. The integrity of RNA was evaluated using the Agilent 2100/4150 (Agilent Technologies, USA), ensuring that all RNA integrity numbers were above 8.0. Purified RNA was sequenced using the NovaSeq X Plus/DNBSEQ T7 (CHI BIOTECH., LTD). Sequencing generated 150-bp strand-specific paired-end reads. Differentially expressed genes (DEGs) were identified with DESeq2 (Version 1.48.1), employing thresholds of |log2(fold change)| ≥1 and an adjusted *P* value < 0.05. Subsequently, the results including heatmap, volcano plot, Gene Ontology (GO) enrichment, Kyoto Encyclopedia of Genes and Genomes (KEGG) pathway mapping, protein–protein interaction map (PPI) network construction and gene set enrichment analysis (GSEA) were visualized using the bioinformatics analysis website (https://bioinformatics.com.cn/).

### *In vivo* implantation of 3D printed scaffolds

Diabetes was induced in 35 rats (6-week-old males, body weight ∼200 g) via a 24 h fast followed via a single intraperitoneal injection of streptozocin (STZ, MCE, NJ, USA, 75 mg kg^−1^). The concentration and treatment time of STZ were adopted from previously published reports [30, 31]. All animal experiments were approved by the Guangzhou Huateng Biopharmaceutical Technology Co., Ltd (B202502-15). Blood glucose was measured after three days, and the rats with levels below 13.5 mM received a second, lower dose of STZ at 20 mg kg^−1^. After a 2-week diabetic stabilization period, 24 successful diabetic rat models were randomly divided into four groups (6 rats per group) based on their body weight. The rats were anesthetized via inhalation of 2% isoflurane (R510-22-10, RWD, Shenzhen, China). A 15 mm longitudinal scalp incision was created along the sagittal suture. Subsequently, two full-thickness 5 mm critical-sized defects were generated in the parietal bones using a sterile dental trephine. These defects in the experimental groups were filled with PH, PHS and PHSN scaffolds, whereas the control group received no implant. All rats were maintained under specific pathogen-free (SPF) conditions, and the skull samples were harvested at 4 and 8 weeks post-implantation for subsequent analysis.

### Micro-CT scanning

Bone regeneration within the defect sites was quantified by micro-computed tomography (micro-CT, NEMO, Jiangsu, China) at a voltage of 80 kV, a current of 100 μA and a voxel size of 14 μm. The obtained slices were reconstructed into 3D images with the accompanying software. The regenerated bone volume (BV), bone volume/total volume (BV/TV) and bone mineral density (BMD) were then evaluated through 3D bone morphometric analysis.

### HE and Masson staining

The skull defect tissues were fixed in 4% PFA (paraformaldehyde) for 48 h at 4°C, followed by decalcification in 10% EDTA (#G1105, Servicebio, Wuhan, China) for 4 weeks. After embedding in paraffin, sections of approximately 5 μm in thickness were prepared. Then these sections were stained with H&E (BASO, BA4025, Zhuhai, China) and Masson’s staining (BASO, BA4079B, Zhuhai, China).

### Immunohistochemistry and immunofluorescence staining

Firstly, paraffin-embedded tissue sections of skull samples were deparaffinized in xylene for 10 min, and then rehydrated through a graded ethanol series (100% to 75%, 5 min each) sequentially. To unmask antigens, the sections were treated with citrate buffer (pH 6.0) (P0081, Beyotime, China) at 58°C overnight. Then the slices were blocked with 3% BSA. Primary antibodies against CD206 (ab300621, Abcam), iNOS (ab283655, Abcam), F4/80 (ab6640), ALP (HUABIO, PSH17-83, Hangzhou, China), OCN (HUABIO, ER1919-20, Hangzhou, China) and CD31 (Servicebio, Wuhan, China) were incubated with the tissue sections overnight at 4°C in the dark. Following this, the sections were exposed to the secondary antibodies, and then the DAB kit (PK1006, Proteintech, Wuhan, China) was used to develop immunostaining. The changes in expression were observed and photographed under a microscope.

### Statistical analysis

Data were analyzed using Origin 2024 and GraphPad Prism 10.0. One-way ANOVA was followed by Dunnett’s test was adopted for single-factor data; two-way ANOVA was used for two-factor (treatment + time point) data. Values are expressed as the mean ± SD. Statistical significance was set at *P < 0.05, **P < 0.01, ***P < 0.001 and ****P < 0.0001.

## Results

### Characterization of PHSN

In this study, both PHS and PHSN scaffolds underwent hydrogel attachment (Figure 2A). Scanning electron microscopy (SEM) analysis revealed that the surfaces of PH, PHS and PHSN scaffolds contained numerous voids, indicating microporous structure of all scaffolds (Figure 2B). All the scaffolds were observed via micro-CT scanning (Figure S1A), and the pore size analysis showed that the mean pore size of PH was 0.3292 ± 0.028 mm, PHS was 0.2951 ± 0.021 mm and PHSN was 0.295 ± 0.0198 mm (mean ± SD, *n* = 3) (Figure S1B). The porosity analysis revealed that the mean porosity of PH was 51.74% ± 1.318, while PHS was 52.26% ± 2.387 and PHSN was 50.60% ± 1.124 (mean ± SD, *n* = 3) (Figure S1C). Meanwhile, confocal microscopy scanning results confirmed that SupGels were successfully coated onto PHS and PHSN scaffolds (Figure S1D). Energy dispersive spectroscopy (EDS) analysis demonstrated that carbon (C), calcium (Ca), oxygen (O) and phosphorus (P) elements were uniformly distributed across these scaffolds. Additionally, sodium (Na) and chlorine (Cl) elements were detected in the PHS scaffolds, and sulfur (S) was also detected in the PHSN scaffold, along with sodium (Na) and chlorine (Cl) (Figure 2C). These results confirm that naringin and SupGels have been successfully loaded onto the surfaces of PHS and PHSN scaffolds. The contact angle analysis showed that the contact angles of PHS (77.63°) and PHSN scaffolds (63.4°) were largely reduced compared to the PH scaffold (98.14°), which suggests that the incorporation of SupGels enhances the hydrophilicity of these scaffolds (Figure 2D and J). Firstly, the FTIR results demonstrated that SupGels was fabricated successfully (Figure S1E). Furthermore, the FT-IR spectra reveal that the PHSN scaffold exhibits distinct absorption bands at 1035.6, 1452.15, 1749.13 and 2933.22 cm⁻¹, which are assigned to the vibrational modes of C = O, C–H, as well as symmetrical and asymmetrical stretching vibrations of C–O–C. Naringin displays characteristic peaks at 2933.22, 1452.15 and 1035.6 cm⁻¹, associated with C–H bonds and the asymmetrical stretching vibration of C–O–C. SupGels show characteristic peaks at 1749.13, 1452.15 and 1035.6 cm⁻¹, corresponding to C–H and C = O bonds, as well as the asymmetrical stretching vibration of C–O–C. Overall, these results indicate the successful integration of naringin and SupGels onto the surface of PH scaffolds (Figure 2E). Moreover, the degradation of scaffolds results indicated that the PHS and PHSN scaffolds exhibited significantly faster degradation performance compared to the PH scaffolds (Figure 2F). Meanwhile, the mechanical testing results demonstrated that the mechanical behavior of the scaffold substrate remains nearly unchanged after hydrogel coating (Figure S1F). The release kinetics of the scaffolds revealed that naringin was released rapidly during the first 7 days, followed by a sustained slow release that extended up to 15 days (Figure 2G). The results of analyzed by ICP-OES demonstrated that the highest release profiles for both calcium and phosphorus were observed in the PHSN scaffold, while the lowest release profiles were observed in the PH group (Figure 2H and I).

**Figure 2:**
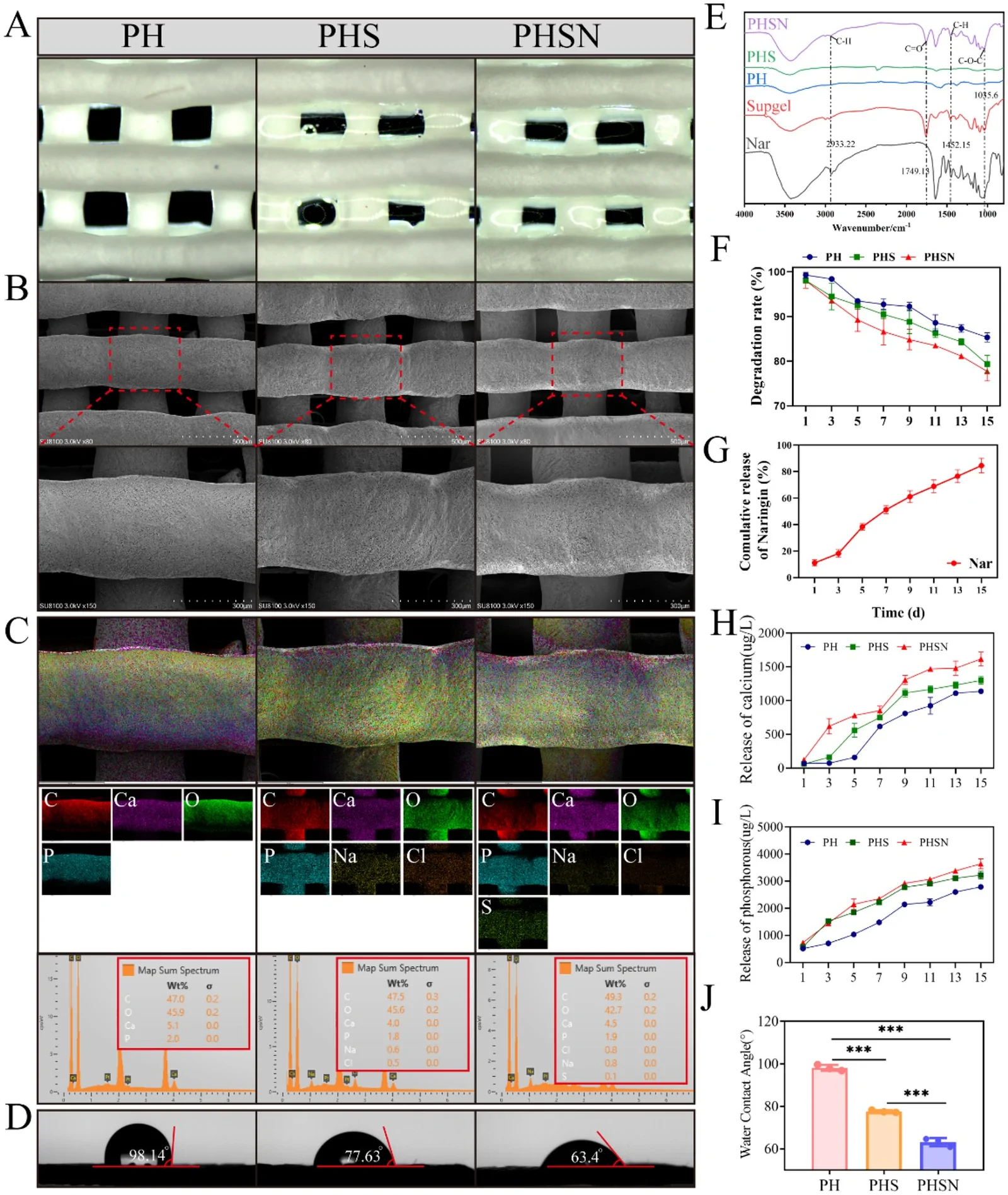
Characterization of the different scaffolds. (**A**) Morphological view. (**B**) SEM observation. (**C**) EDS analysis. (**D**) Water contact angle measurement. (**E**) FT-IR spectra analysis. (**F**) Degradation rate. (**G**) Cumulative release of naringin. (**H**) Release of calcium. (**I**) Release of phosphorous. (**J**) Statistics of water contact angle. Values are expressed as the means ± SD. One-way ANOVA followed by Dunnett’s test was adopted for single-factor data; two-way ANOVA was used for two-factor (treatment + time point) data. *P < 0.05, **P < 0.01, ***P < 0.001.

### *In vitro* biocompatibility of PHSN

Next, we tested the biocompatibility of the scaffolds through cell culture experiments. The CCK-8 assays demonstrated that at a concentration of 100 µg/mL, naringin could enhance the proliferation and differentiation of BMSCs (Figure 3B). Following exposure to the extract solution for 1, 2 and 3 days, Live/Dead staining indicated that no cytotoxicity was observed in any of the treatment groups (Figure 3A and C). For the HUVECs, Live/Dead staining also demonstrated that no cytotoxicity was observed in any of the treatment groups (Figure S2A and C). Furthermore, Live/Dead staining revealed the presence of some dead cells on the PH and PHS scaffolds, whereas almost no dead cells were detected on the PHSN scaffold (Figure 3D and E), suggesting that the PHSN scaffold exerted no adverse effects on the cells. Additionally, phalloidin staining demonstrated that BMSCs were able to attach and proliferate well on all three scaffolds; notably, more cells attached to the PHSN scaffold compared with the PH and PHS scaffolds (Figure 3F).

**Figure 3:**
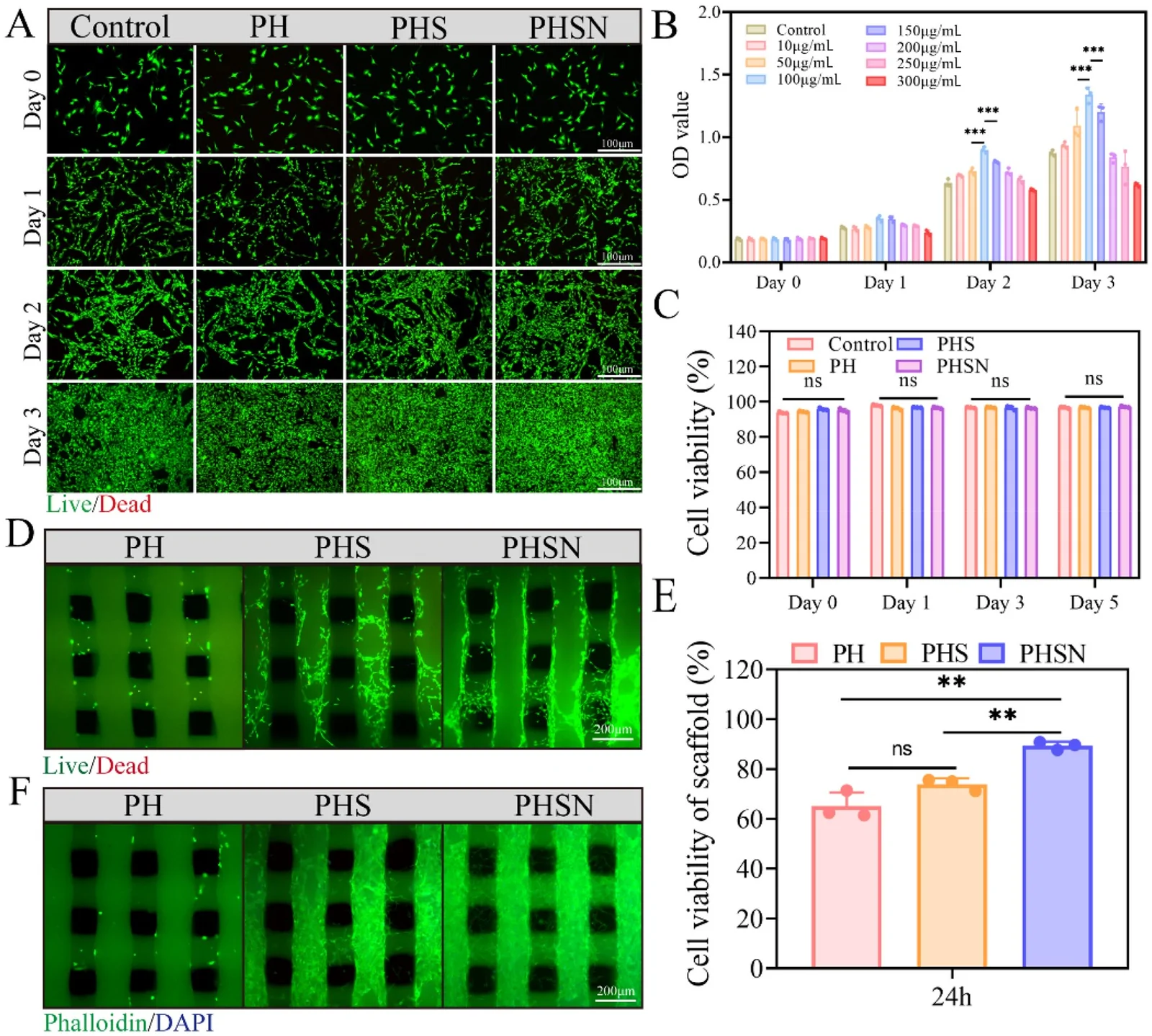
Cytotoxic effects of naringin and biocompatibility performance of different scaffolds. (**A**) Calcein/PI staining. (**B**) CCK-8 assay. (**C**) Statistics of cell viability. (**D**) Calcein/PI staining of the scaffolds. (**E**) Statistics of cell viability on those scaffolds. (**F**) Phalloidin staining. Values are expressed as the means ± SD. One-way ANOVA followed by Dunnett’s test was adopted for single-factor data; two-way ANOVA was used for two-factor (treatment + time point) data. *P < 0.05, **P < 0.01, ***P < 0.001.

### *In vitro* immunomodulatory effects of PHSN

M2 macrophages play a crucial role in promoting bone repair during the early stages. Our data from the *in vitro* co-culture of RAW264.7 cells for 48 h with conditional medium from different scaffolds indicated the immunomodulatory capacity of our scaffolds. Data showed that the iNOS expression was significantly increased in RAW264.7 cells following lipopolysaccharide (LPS) stimulation, suggesting macrophage polarization towards the M1 phenotype. For CD206, a marker indicative of M2 macrophages, immunofluorescence staining revealed a significantly higher proportion of M2 macrophages in the PHSN group compared with both the PH and PHS groups (Figure 4A and C). This predominance is associated with the effects of naringin released from the SupGels, which were coated onto the scaffolds. In contrast, treatment with PH, PHS and PHSN resulted in low expression levels of iNOS, indicating that none of the scaffolds induced the inflammatory response (Figure 4B and D). Furthermore, the gene expression analysis demonstrated that M2 macrophage-related genes (CD206, Arg-1, IL-10) were upregulated (Figure 4F), while the M1 macrophage-related gene (iNOS) was also upregulated (Figure 4E).

**Figure 4:**
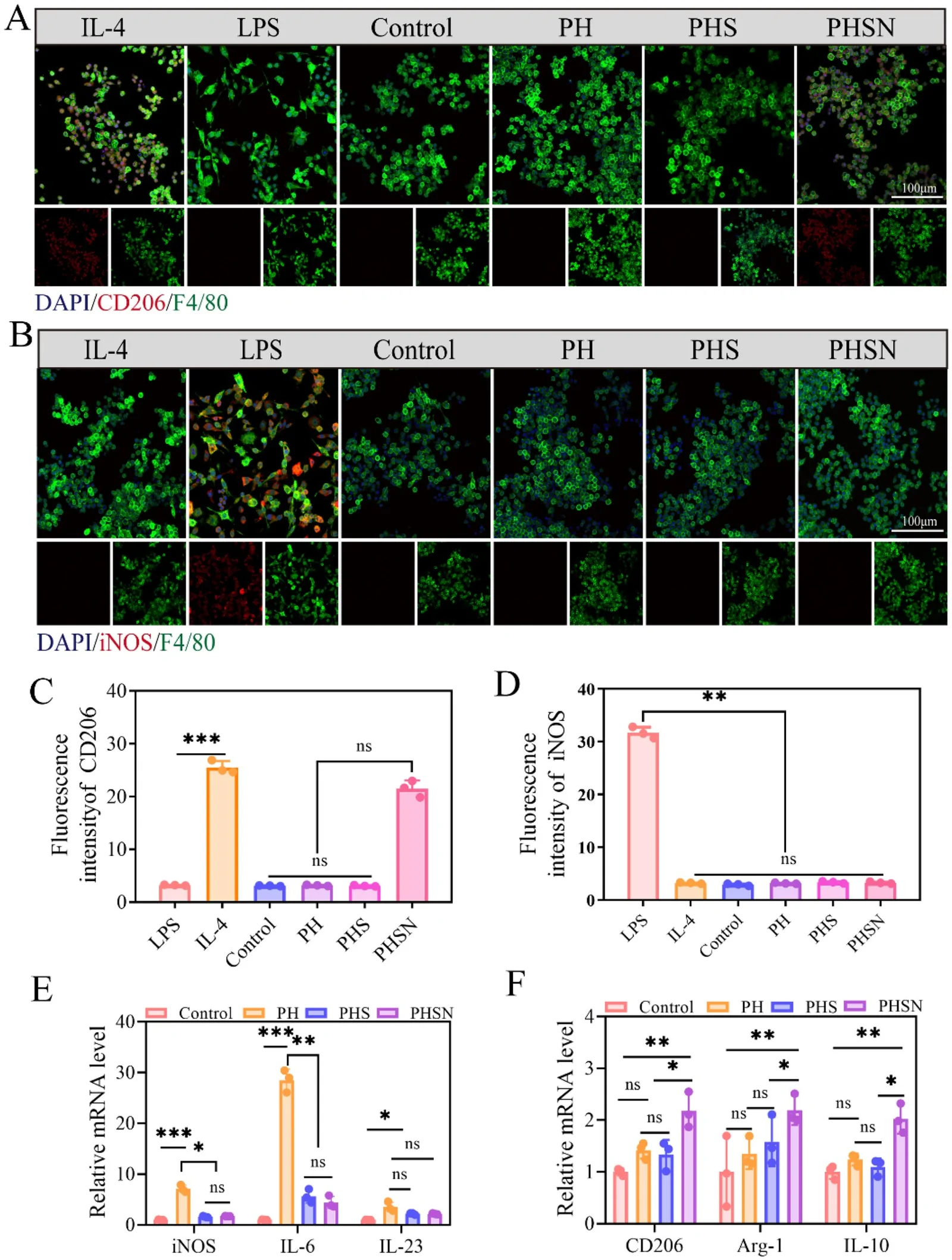
*In vitro* responses of macrophages to scaffolds. (**A**) Immunofluorescent staining of the M2 marker (CD206). (**B**) Immunofluorescent staining of the M1 marker (iNOS). (**C**) Statistics of the CD206 staining results. (**D**) Statistics of the iNOS staining result. (**E**) mRNA expression levels of genes iNOS, IL-6 and IL-23. (**F**) mRNA expression levels of genes CD206, Arg-1 and IL-10. Values are expressed as the means ± SD. One-way ANOVA followed by Dunnett’s test was adopted for single-factor data; two-way ANOVA was used for two-factor (treatment + time point) data. *P < 0.05, **P < 0.01, ***P < 0.001.

### *In vitro* migration and angiogenic capacity of PHSN

A wound scratch assay was used to evaluate the migration effects of cells. The results indicated that treatment with PH, PHS and PHSN scaffolds, which can promote the migration of HUVECs. Specifically, the PHSN scaffold significantly enhanced the migration ability of HUVECs compared with both PH and PHS (Figure 5A and B). Tube formation assays further indicate that treatment with PH, PHS and PHSN scaffolds can enhance the tube-forming ability of HUVECs, and the PHSN scaffold exhibited superior angiogenic activity compared with PH and PHS (Figure 5C–E). Moreover, the PHSN scaffolds also exhibited superior angiogenic activity compared with naringin (Figure S2B, D and E). Collectively, these results indicated that PHSN could significantly promote both angiogenesis and migration of HUVECs.

**Figure 5:**
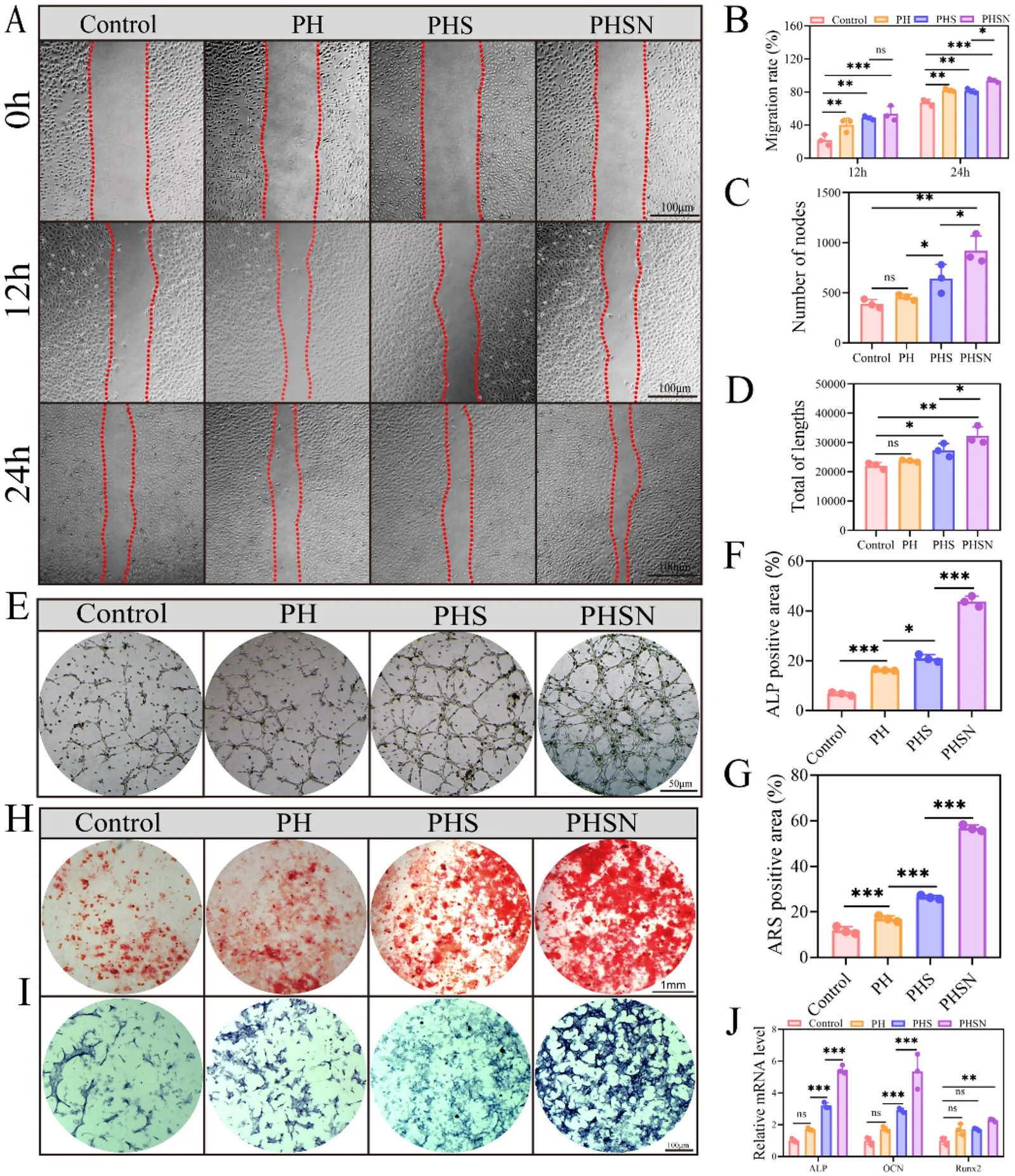
Migration, angiogenic capacity and osteogenesis performances of different scaffolds *in vitro*. (**A**) Migration of HUVECs. (**B**) Statistics of migration rate. (**E**) Tube formation assay. (**C and D**) Statistics of the number of nodes and total tube length. (**F**) Statistical of ALP-positive area. (**H**) ARS-staining. (**G**) Statistics of ARS positive area. (**I**) ALP staining. (**J**) mRNA expression levels of the genes ALP, OCN and Runx2. Values are expressed as the means ± SD. One-way ANOVA followed by Dunnett’s test was adopted for single-factor data; two-way ANOVA was used for two-factor (treatment + time point) data. *P < 0.05, **P < 0.01, ***P < 0.001.

### *In vitro* osteogenic differentiation capacity of PHSN

The osteogenesis capacity of BMSCs was evaluated via ALP activity staining and ARS mineralization staining on days 7 and 14. The ALP staining results indicated that after treatment with PH, PHS and PHSN, cells in all groups exhibited varying degrees of bluish-purple precipitates. Notably, the PHSN group demonstrated significantly deeper and more extensive bluish-purple precipitation compared with the PH and PHS groups (Figure 5F and I). Furthermore, ARS staining was performed to detect the effect of PHSN on mineralized matrix formation of BMSCs during late-stage osteogenesis. The findings revealed that the PHSN group exhibited a greater quantity and larger size of mineralized nodules compared to the other groups (Figure 5G and H). Yet compared with PHSN, scaffolds (no co-culture with macrophage) or naringin treatment with BMSCs exhibited inferior osteogenic capacity (Figure S3A and C). Additionally, PHSN significantly upregulated the expression of osteogenic genes ALP, OCN and Runx2 (Figure 5J).

### *In vivo* cranial defect repair capacity of PHSN

Given the promising *in vitro* performance of PHSN observed *in vitro*, a critical-sized calvarial defect model was generated in diabetic rats to comprehensively evaluate its bone regeneration potential (Figure 6A). Following a 4-week implantation period, micro-CT scans revealed that the PHSN group achieved significantly superior bone regeneration, specifically, it exhibited the highest values in regenerated bone volume, bone volume fraction and bone mineral density among all groups (PHSN vs PH and PHS). After 8 weeks of implantation, the PHSN group continued to outperform the PH and PHS groups in all assessed indicators of bone regeneration (Figure 6B–E). These results are consistent with research results *in vitro*. H&E staining revealed that significantly less new bone formation at the defect edge in the control group. Conversely, the defect site in the PHSN group showed abundant new trabecular bone formation, along with reduced inflammation and numerous new capillaries (Figure 6F). Meanwhile, Masson’s staining indicated that, compared with the PH and PHS scaffolds, the PHSN-treated group exhibited extensive bright red mineralized bone matrix surrounded by blue-stained osteoid, suggesting greater deposition of collagen fibers within the diabetic bone defect area (Figure 6G).

**Figure 6:**
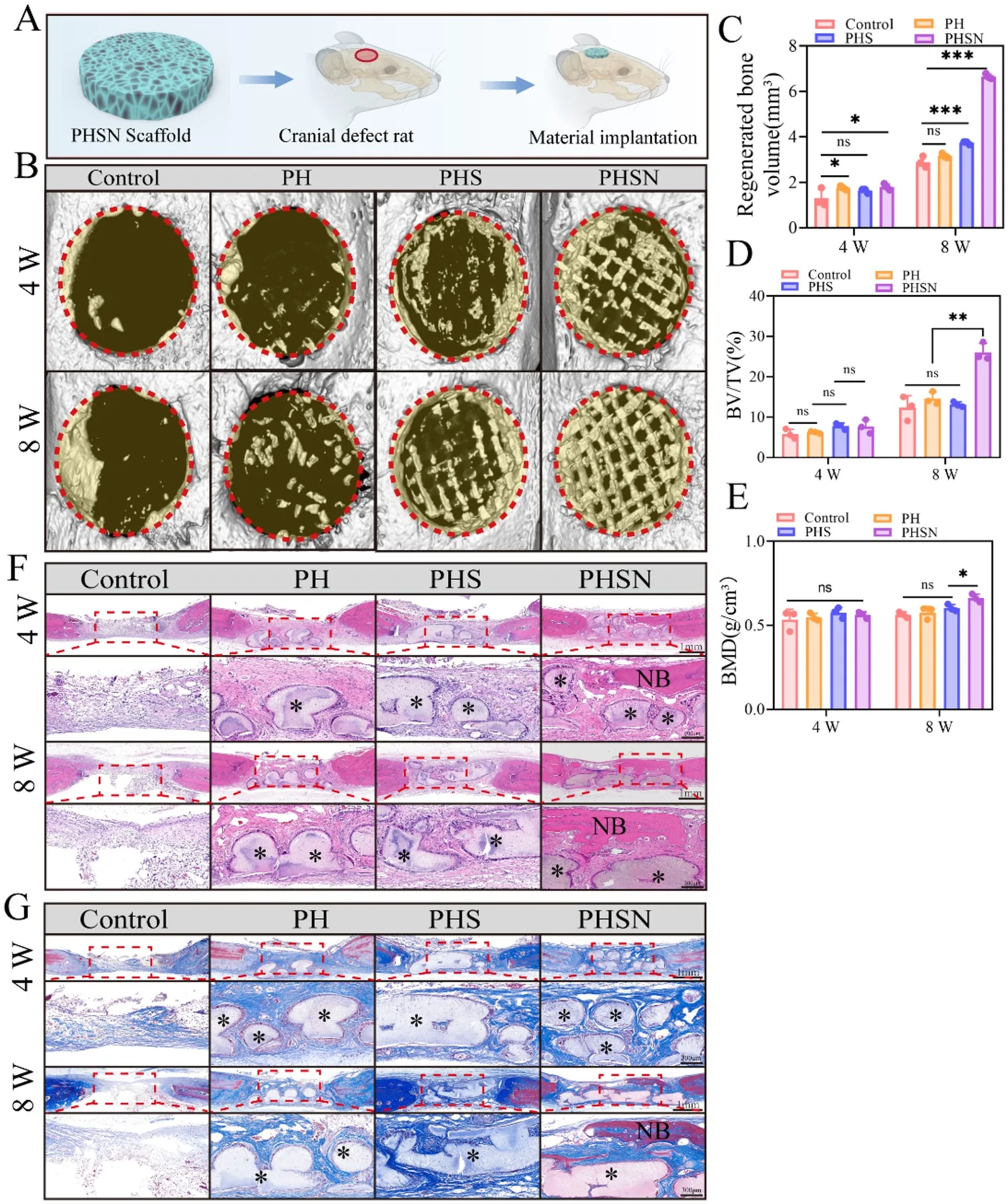
*In vivo* osteogenic effects of different scaffolds. (**A**) Schematic diagram of different scaffold implantation. (**B**) Representative 3D micro-CT images at 4 weeks and 8 weeks post-operation. (**C**) Statistics of regenerated bone volume. (**D and E**) Statistics of BV/TV and BMD. (**F**) HE staining. (**G**) Masson’s staining. NB, new bone. *, denote the 3D-printed scaffolds. Values are expressed as the means ± SD. One-way ANOVA followed by Dunnett’s test was adopted for single-factor data; two-way ANOVA was used for two-factor (treatment + time point) data. *P < 0.05, **P < 0.01, ***P < 0.001.

### *In vivo* osteogenic differentiation and osteoclast formation

The levels of alkaline phosphatase (ALP) and osteocalcin (OCN) in the bone defect areas of diabetic rats were assessed through immunohistochemical analysis to further confirm the osteogenic differentiation-promoting effect of PHSN *in vivo*. ALP staining at 4 and 8 weeks post-implantation revealed significantly higher expression levels in the PHSN group compared with the PHS, PH and control groups (Figure 7A and B). Similarly, OCN staining demonstrated markedly elevated expression levels in the PHSN groups relative to the PHS, PH and control groups at both 4 and 8 weeks post-implantation (Figure 7C and D). Bone remodeling relies on a balance between osteoblast-mediated formation and osteoclast-mediated resorption. Consequently, TRAP staining is commonly used to identify and quantify osteoclast activity. TRAP staining indicated a greater presence of red color in the control and PH groups relative to the PHS and PHSN groups at 4 weeks. By 8 weeks post-implantation, TRAP staining showed almost no red staining in the PHSN group, whereas the control, PH and PHS groups exhibited some remaining red staining (Figure 7E and F).

**Figure 7:**
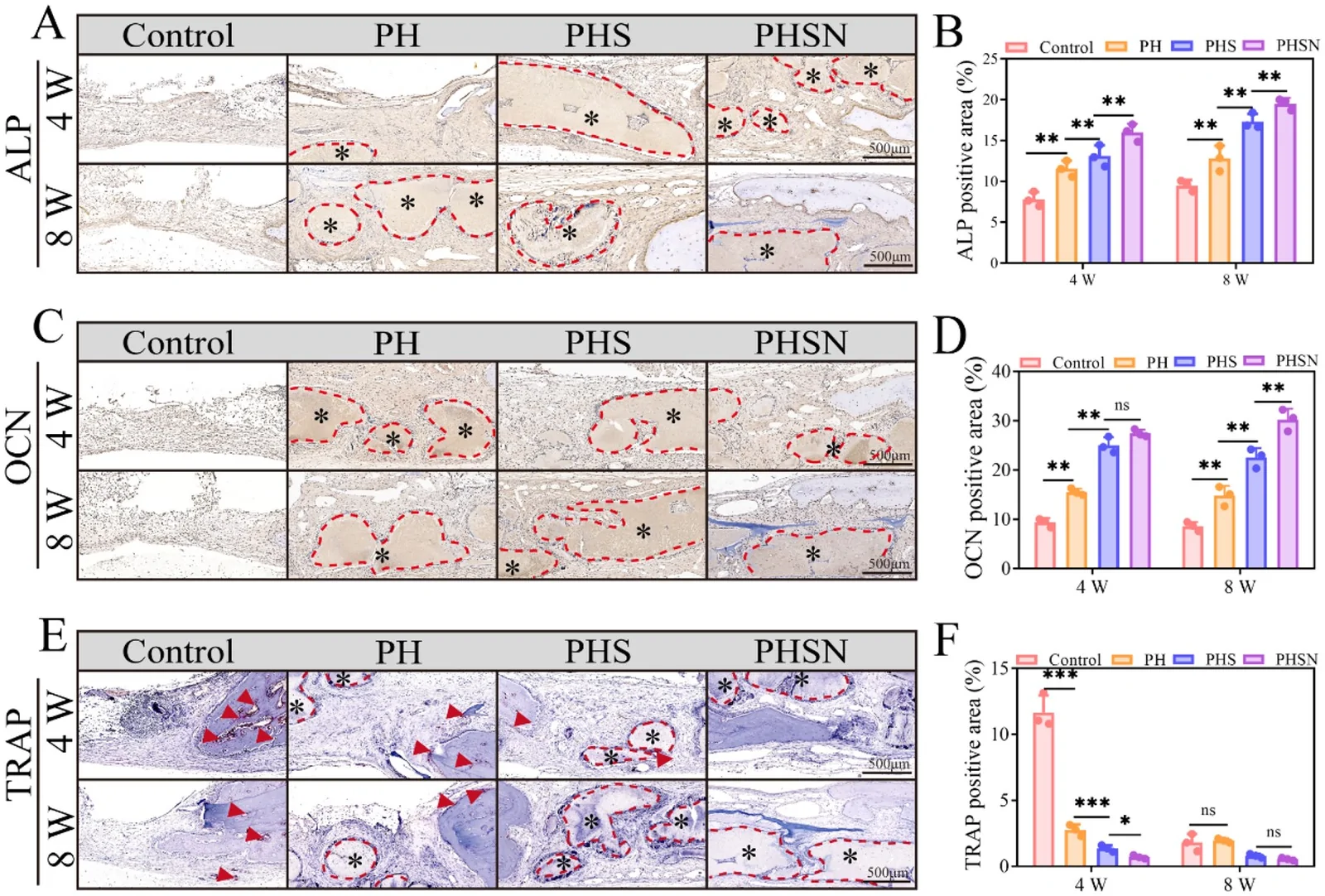
*In vivo* osteogenic and osteoclastogenic performances of different scaffolds under diabetic conditions. (**A**) Immunohistochemical staining of ALP. (**B**) Statistics of ALP-positive staining area. (**C**) Immunohistochemical staining of OCN. (**D**) Statistics of OCN-positive staining area. (**E**) TRAP staining. (**F**) Statistics of TRAP-positive area. ▲, staining positive area. *, denote the 3D-printed scaffolds. Values are expressed as the means ± SD. One-way ANOVA followed by Dunnett’s test was adopted for single-factor data; two-way ANOVA was used for two-factor (treatment + time point) data. * *P *< 0.05, ** *P *< 0.01, *** *P *< 0.001.

### *In vivo* immune and angiogenic regulation

*In vivo* immunofluorescence staining of CD206 demonstrated that the PHSN group exhibited significantly higher CD206 expression relative to the control, PH and PHS groups at 4 weeks. However, at 8 weeks, the expression of CD206 decreased in all treatment groups, yet the PHSN group maintained the highest expression among the treatment groups (Figure 8A and B). Immunofluorescence staining for iNOS indicated that the control and PH groups had higher iNOS expression, while the PHS and PHSN groups displayed the lowest expression levels at both 4 and 8 weeks. Notably, the expression of iNOS was significantly lower in the PHSN group (Figure 8C and D). Additionally, the results of CD31 immunofluorescence staining exhibited a significantly greater number of newly formed blood vessels in the PHSN group (Figure 8E and F).

**Figure 8:**
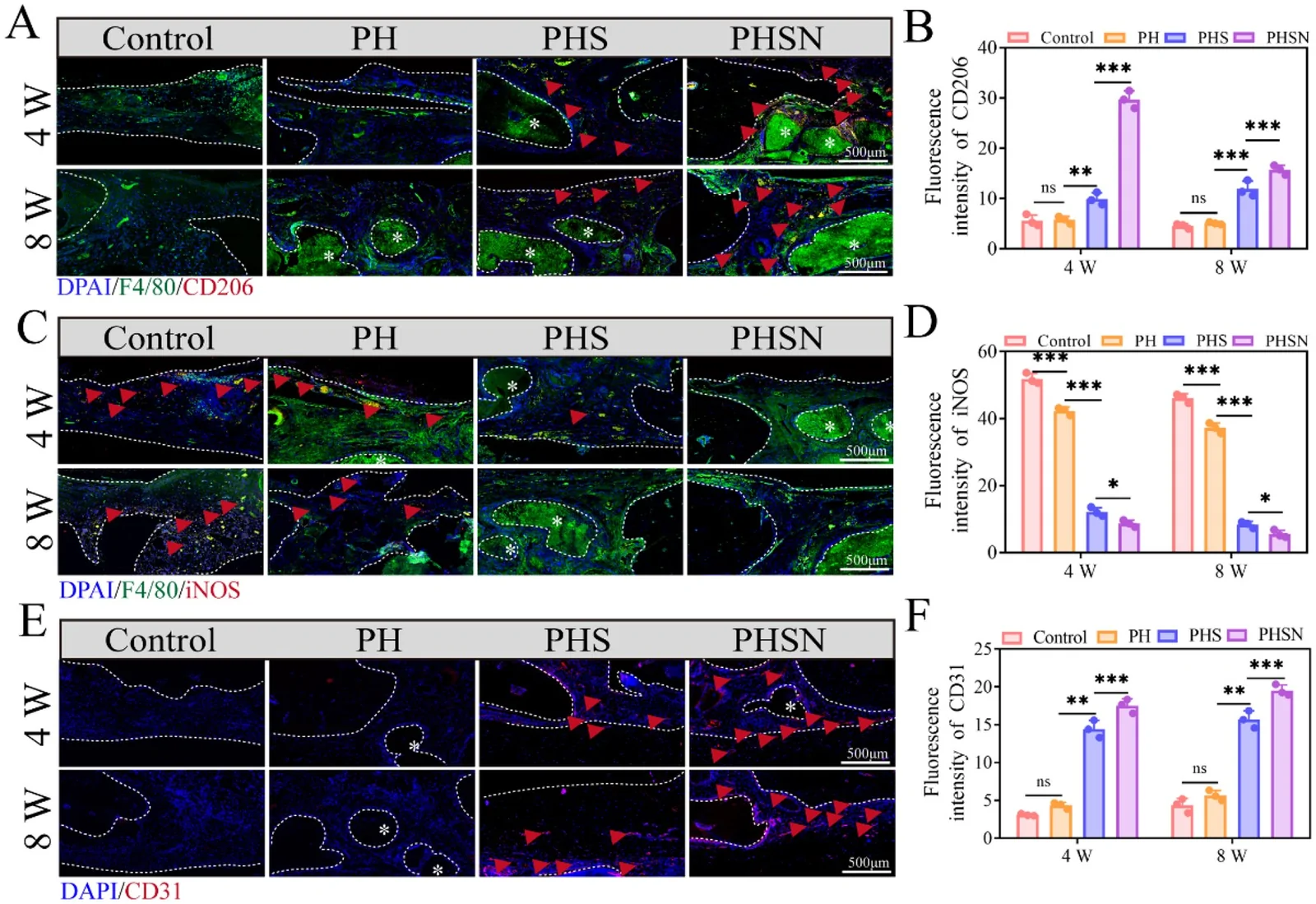
*In vivo* immunomodulatory and angiogenic bioactivities of these scaffolds under diabetic conditions. (**A**) Immunofluorescent staining of M0 and M2 markers (F4/80 and CD206). (**B**) Statistics of the CD206 staining results. (**C**) Immunofluorescent staining of M0 and M1 markers (F4/80 and iNOS). (**D**) Statistics of the iNOS staining results. (**E**) Immunofluorescent staining of angiogenic marker (CD31). (**F**) Statistics of the CD31 staining result. ▲, staining positive area. *, denotes the 3D-printed scaffolds. Values are expressed as the means ± SD. One-way ANOVA followed by Dunnett’s test was adopted for single-factor data; two-way ANOVA was used for two-factor (treatment + time point) data. * *P *< 0.05, ** *P *< 0.01, *** *P *< 0.001.

### Mechanism of immunomodulatory effects of PHSN

To investigate the mechanism underlying immunomodulatory effects of PHSN, *in vitro* transcriptomic analysis was performed using RAW264.7 cells. Pearson correlation analysis showed satisfactory uniformity and reliability within each group (Figure 9A). Volcano plots and heatmaps (Figure 9B and C) revealed extensive differential gene expression induced by PHSN, with 272 genes upregulated and 81 genes downregulated. Gene ontology (GO) database analysis showed that the up-regulated genes related to cell motility and immunomodulation were significantly enriched in the PHSN group compared with the control group (Figure 9G). Accordingly, these significantly up-regulated genes were divided into three typical categories, including macrophage polarization (Figure 9D), cell migration (Figure 9E) and cell differentiation (Figure 9F). KEGG analysis showed that PHSN group could enhance hematopoietic cell lineage signaling pathway and stimulate the immune responses. Additionally, increased activity was observed in the JAK/STAT, MAPK and PI3K/AKT signaling pathways (Figure 9H). Protein–protein interaction network analysis further exhibited the protein network associated with the JAK/STAT, MAPK and PI3K/AKT signaling pathways (Figure 9I). The GSEA analysis further highlighted the JAK/STAT signaling pathway was significantly enriched (Figure 9J). The Western blot analysis results confirm that naringin could trigger activation of the JAK/STAT signaling pathway by increasing the phosphorylation levels of the JAK2, STAT3 and STAT6 (Figure 9K).

**Figure 9:**
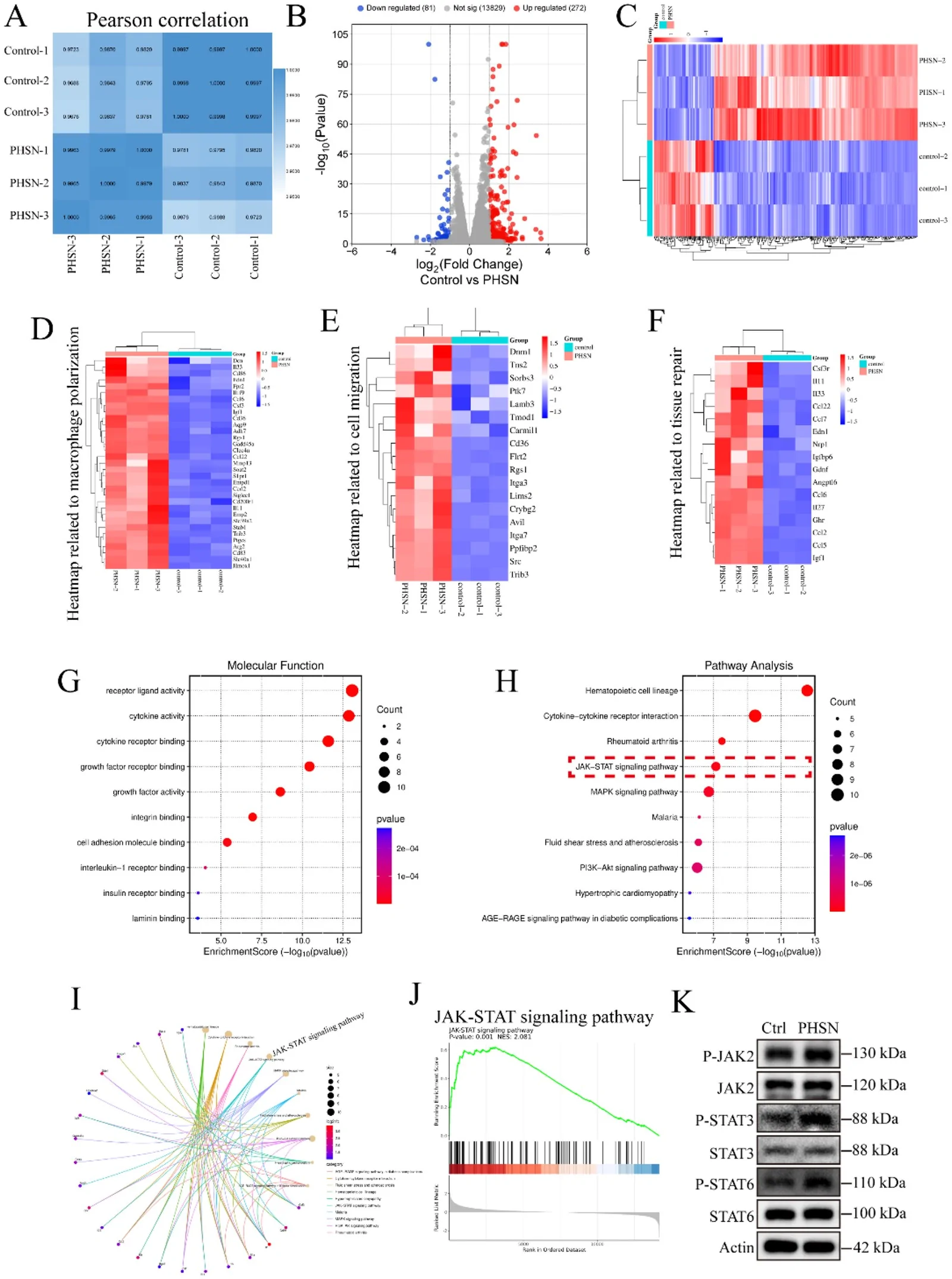
Transcriptome sequencing and signaling pathway analysis. (**A**) Pearson correlation analysis between samples. (**B**) Volcano plot of differentially expressed genes. (**C**) Heatmap and hierarchical clustering analysis of differentially expressed genes. (**D–F**) Heatmap analysis of differentially expressed genes related to macrophage polarization, cell migration and cell differentiation. (**G**) Enriched upregulated go terms of control versus PHSN. (**H**) Enriched KEGG pathways of control versus PHSN. (**I**) Protein–protein interaction network analysis of differentially expressed genes. (**J**) GSEA analysis of JAK/STAT signaling pathway. (**K**) Western blot analysis of JAK/STAT signaling pathway-related proteins.

## Discussion

In diabetic patients, alterations in the local microenvironment following bone defect significantly influences repair efficacy. The hyperglycemic environment can impair angiogenesis and induce persistent inflammatory responses, severely hindering the bone defect repair process [32]. Naringin plays a critical role in the regulation of immunity and bone repair [33]. Naringin-derived bioink for 3D bioprinting enhances the efficiency of cartilage defect repair [34]. 3D-printed polymeric-based scaffold coated with bioceramics and naringin has been demonstrated to promote dental pulp regeneration [35]. However, the immunomodulatory mechanism of naringin and its corresponding downstream effects after immune regulation remain unclear. 3D-printed PLGA/HA scaffolds exhibit significant potential in bone tissue engineering; such as possessing 3D structure for cell growth as well as good mechanical properties [36]. But the bioactivity of PLGA/HA composite scaffolds is constrained due to insufficient bioactive sites on their surface. Meanwhile, Poor hydrophilicity reduces the tissue compatibility of PLGA scaffolds [37].

Our research focused on the reparative effects of the natural flavonoid naringin and the combination of SupGels hydrogel with PLGA/HA materials on diabetic bone defects. At a concentration of 200 μg/mL, naringin promotes both the proliferation and osteogenic differentiation of human amniotic fluid-derived stem cells (hAFSCs) [38]. Additionally, studies using rat models indicate that 10 μg/mL naringin most effectively stimulates osteocalcin production [39]. In our research, at a concentration of 100 μg/mL, naringin can promote cell proliferation, consistent with previous reports [38, 39]. SupGels possess excellent drug loading capacity [27]. In our study, naringin and SupGels were successfully coated onto the surface of the PLGA/HA scaffold, allowing naringin to be slowly released from the scaffold. Furthermore, the PHSN scaffold exhibited favorable degradation characteristics, releasing Ca^2+^ and P ions (Figure 2H and I), displayed superior hydrophilicity, all of which are beneficial for subsequent cell proliferation. Moreover, the incorporation of SupGels enhanced the cell recruitment capacity (Figure 3F) and cell survival capability (Figure 3A and D) of the PHSN scaffold. Collectively, these results fully demonstrated the biocompatibility of the PHSN scaffold.

Macrophages play a crucial role in bone repair after injury [40]. In the initial stage of bone repair, macrophages polarize towards the M1 phenotype via the classical pathway. M1 macrophages produce inflammatory cytokines (TNF-α, IL-6 and IL-1β) to suppress osteogenesis [41]. Therefore, regulating the shift of macrophage phenotype from M1 to M2 is critical for enhancing bone repair during the initial regenerative phase [42]. In our study, PHSN promoted M2 macrophage polarization through upregulation of CD206, Arg-1 and IL-10 (Figure 4A and F). Conversely, PHSN suppressed M1 polarization by downregulating the expression of iNOS, IL-6 and IL-23 (Figure 4B and E). Thus, these results demonstrate that PHSN induces macrophage polarization toward the M2 phenotype, thereby laying foundation for subsequent bone repair. Angiogenesis and osteogenesis are both critical in diabetic bone repair [43]. In normal bone repair, angiogenesis and osteogenesis are interdependent; angiogenesis not only supplies essential oxygen and nutrients to bone tissue but also promotes the differentiation of osteoblasts and bone formation through the secretion of various growth factors such as VEGF and BMP2 [44]. However, under diabetic conditions, hyperglycemia and oxidative stress disrupt this coupling, resulting in reduced angiogenesis and impaired bone formation [45]. In the tube formation assay, conditioned media from the PHSN scaffolds significantly promote the migration and tube formation of HUVECs. Furthermore, it also enhanced the osteogenesis of BMSCs by upregulating the gene expression of alkaline phosphatase (ALP), osteocalcin (OCN) and runt-related transcription factor 2 (Runx2). Thus, we speculate that the osteogenic and angiogenic effects of PHSN are mediated through the activation of cytokine signaling pathways in macrophages. *In vivo*, micro-CT analysis, H&E staining and Masson’s staining demonstrated that PHSN exhibited excellent osteogenic repair capacity. Meanwhile, the PHSN group exhibited the highest expression of osteogenesis-related markers ALP and OCN, while TRAP staining indicated that PHSN suppressed osteoclast formation. In addition, immunostaining for macrophage polarization markers showed that PHSN induced M2 polarization (Figure 8A) and suppressed M1 polarization (Figure 8C). CD31 staining indicated that PHSN promotes angiogenesis (Figure 8E), which may be attributed to the application of SupGels and naringin. Collectively, these results are consistent with the *in vitro* cellular experiments, demonstrating that PHSN can facilitate vascular regeneration and accelerate bone repair by regulating macrophage polarization.

To investigate the underlying regulatory mechanisms of naringin on macrophage polarization, RNA-sequencing (RNA-seq) was performed to analyze the gene expression alterations in macrophages treated with the PHSN group and control group. The macrophage phenotype [46] and cell migration [47] play a crucial role in regulating bone defect repair. Our results showed that upregulated genes were associated with M2 macrophage polarization (Figure 9D), cell migration (Figure 9E) and tissue repair (Figure 9F), demonstrated that PHSN scaffold can drive macrophages into the M2 type and facilitate cell migration and tissue repair. In addition, KEGG enrichment analysis revealed that JAK/STAT signaling pathway was closely associated with macrophage polarization and cell migration (Figure 9H–J).

The JAK/STAT signaling pathway is critically involved in the regulation of macrophage [48]. According to previous studies, there are numerous key factors which can regulate JAK/STAT signaling pathway, including Janus kinase 2 (JAK2), phosphorylated JAK2, STAT3, phosphorylated STAT3, STAT6, phosphorylated STAT6 [43]. Western blot assay results showed that in PHSN group, the protein levels of phosphorylated JAK2, phosphorylated STAT3 and phosphorylated STAT6 were significantly higher compared with control group. Meanwhile, the AG490 inhibitor was used to verify the regulatory role of PHSN in the JAK/STAT signaling pathway. The results showed that upon treated with AG490, the expression level of p-JAK2, p-STAT3, p-STAT6 protein were downregulated, and the number of M2 macrophages was diminished, osteogenic function was compromised (Figure S4). The elevated expression of P-JAK2, P-STAT3 and P-STAT6 in the PHSN group verifies that JAK/STAT signaling pathway could be a key pathway by which PHSN promote M2 macrophage polarization (Figure 10).

**Figure 10:**
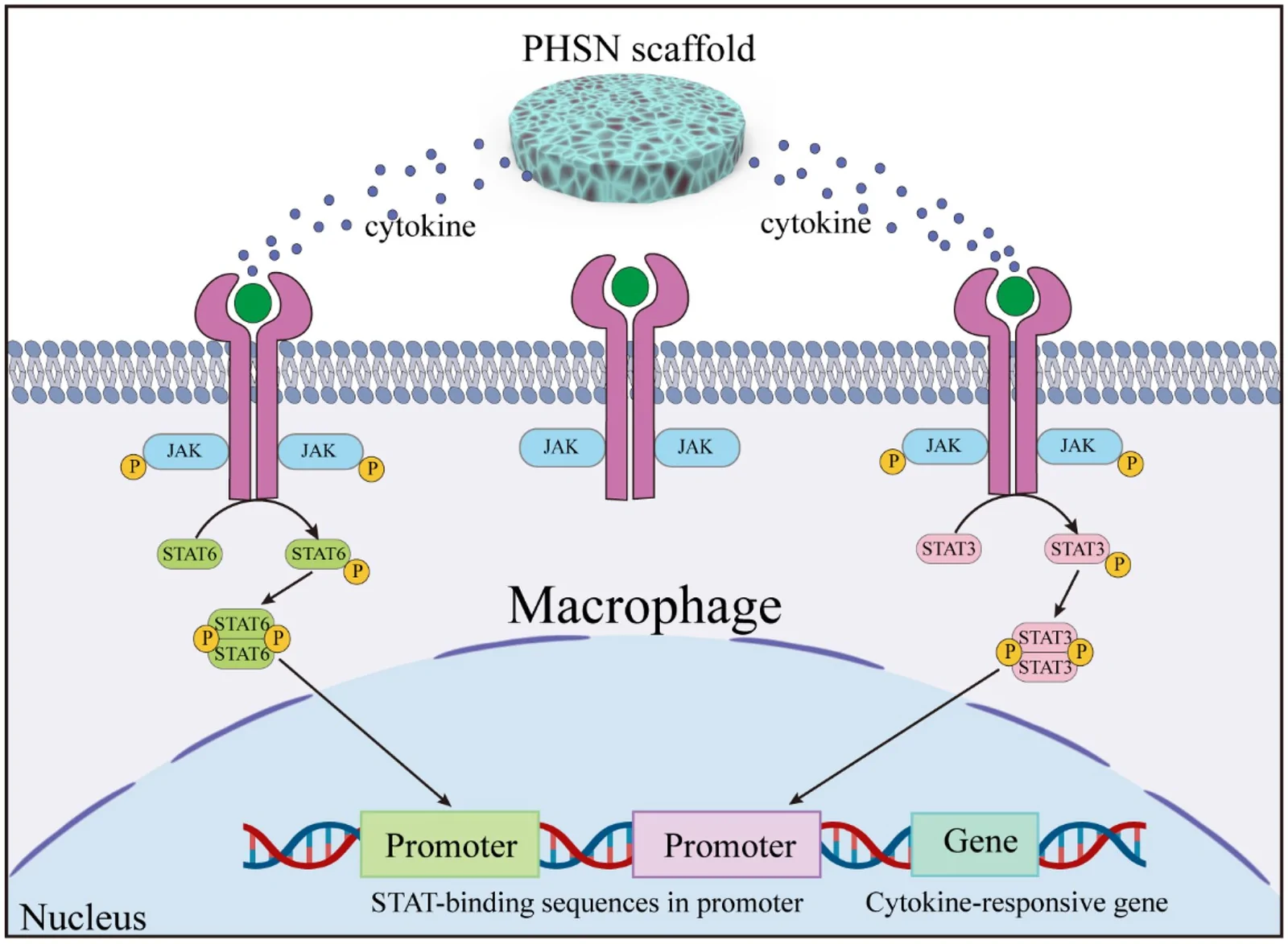
Schematic illustration of PHSN scaffolds promotes M2 macrophage polarization via JAK/STAT signaling pathway.

In summary, the innovated PHSN scaffold developed in this study exerts a significant regulatory effect on regulating macrophage polarization both *in vitro* and *in vivo*, effectively facilitating cranial defects repair in diabetic rats. However, our study has the following limitations: Firstly, the diabetic animal model was induced by STZ, cannot simulates the pathological characteristic of clinical type 2 diabetes. Secondly, although we have shown that the conditioned medium from PHSN-treated macrophages can effectively promote osteogenesis and angiogenesis, further experiments are needed to identify the specific molecules in the conditioned media that play a significant role in this process.

## Conclusion

Diabetes mellitus markedly impairs bone regeneration by dysregulating the immune responses and impairing local angiogenesis. In this study, the PHSN scaffold exhibited multifaceted therapeutic properties, including robust immunomodulatory capacity, excellent biocompatibility and capacity of pro-osteogenesis and pro-angiogenesis. This immunomodulatory function promoted macrophage polarization toward the M2 phenotype via the JAK/STAT signaling pathway, thereby facilitating bone regeneration. In summary, the PHSN scaffold is an innovative biomaterial engineered for diabetic bone repair and provides a novel therapeutic strategy for this challenging condition.

## Supplementary Material

rbag161_Supplementary_Data
