# Supplementary material for: Naringin-based hydrogel decorated 3D printed scaffold modulates immunity and angiogenesis for diabetic bone regeneration
Source: Regen Biomater. 2026 Aug 5;13:rbag161. doi: 10.1093/rb/rbag161 (PMC13627827; doi:10.1093/rb/rbag161)
Supplement: Supplementary file 1 [file rbag161_supplementary_data.docx]

Supplementary Figure S1. Micro-CT scanning of the scaffolds (A). statistical analysis of porosity (B). statistical analysis of pore size (C). FITC staining of SupGel in the scaffolds (D). FTIR analysis of GelMA and AC-β-CD (E). Mechanical testing of the scaffolds (F).

Supplementary Figure S2. Live/Dead staining of Huvec cell (A). Tube formation assay (B). Cell viability analysis of live/dead staining (C). total length and number nodes analysis of tube assay (D, E).

Supplementary Figure S3. ALP staining of osteogenesis culture with scaffold condition medium (A). ALP positive area analysis (B). ALP staining of osteogenesis culture with scaffold-macrophage co-condition medium (C). ALP positive area analysis (D). IL-10 expression level of scaffold-macrophage co-culture condition medium (ELISA assay) (E). TGF-β1 expression level of scaffold-macrophage co-culture condition medium (ELISA assay) (F).

Supplementary Figure S4. Western blot analysis (A). Immunofluorescence staining of macrophage (B). Analysis of fluorescence intensity of CD206 (C). Tube formation assay of HUVEC cells (D). Analysis of Total length and number nodes (E, F). ALP staining for osteogenic differentiation(G). Analysis of ALP staining (H).

Supplementary Figure S5. HE staining (20X, A). *, denote the 3D printed scaffold.
